# Experience of sexual coercion and risky sexual behavior among Ugandan university students

**DOI:** 10.1186/1471-2458-11-527

**Published:** 2011-07-04

**Authors:** Anette Agardh, Karen Odberg-Pettersson, Per-Olof Östergren

**Affiliations:** 1 Social Medicine and Global Health, Department of Clinical Sciences Malmö, Lund University, CRC, Entrance 72, Skåne University Hospital, 205 02 Malmö, Sweden

## Abstract

**Background:**

Growing worldwide evidence shows that the experience of sexual coercion is fairly prevalent among young people and is associated with risky sexual behavior thereafter. The causal mechanisms behind this are unclear but may be dependent on specific contextual determinants. Little is known about factors that could buffer the negative effects of coercion. The aim of this study was to assess the association between the experience of sexual coercion and risky sexual behavior among university students of both sexes in Uganda.

**Methods:**

In 2005, 980 (80%) out of a total of 1,220 students enrolled in Mbarara University of Science and Technology in Uganda participated in a self-administered questionnaire covering socio-demographic and religious factors, social capital, mental health, alcohol use, and sexual behavior. A validated scale of six items was used to assess the experience of sexual coercion. Logistic regression analyses were applied to control for confounders. Potential buffering factors were analyzed by testing for effect modification.

**Results:**

Fifty-nine percent of those who responded had previously had sexual intercourse. Among the male students 29.0%, and among the female students 33.1% reported having had some experience of sexual coercion. After controlling for age, gender, and educational level of household of origin, role of religion and trust in others sexual coercion was found to be statistically significantly associated with previously had sex (OR 1.6, 95% CI; 1.1-2.3), early sexual debut (OR 2.4, 95% CI; 1.5-3.7), as well as with having had a great number of sexual partners (OR 1.9, 95% CI; 1.2-3.0), but not with inconsistent condom use.

Scoring low on an assessment of mental health problems, reporting high trust in others, or stating that religion played a major role in one's family of origin seemed to buffer the negative effect that the experience of sexual coercion had on the likelihood of having many sexual partners.

**Conclusion:**

The findings of this study suggest that the experience of sexual coercion is common among youth/young adults in Uganda and is subsequently associated with risky sexual behavior in both sexes. The existence of individual and contextual factors that buffer the effects mentioned was also demonstrated. In the Ugandan context, this has implications for policy formulation and the implementation of preventive strategies for combating HIV/AIDS.

## Background

Several studies have suggested that the experience of sexual coercion leads to a greater likelihood of risky sexual behavior, such as early sexual debut, many sexual partners, and inconsistent condom use [1-5]. Since such behaviors are the target of prevention efforts against HIV/AIDS, knowledge of the prevalence of sexual coercion, the specific mechanisms through which it is carried out, and its subsequent impact on risky sexual behaviors are especially relevant in communities where the disease continues at epidemic proportions. Despite a substantial fall in the prevalence of HIV/AIDS from 15% in 1991 [6] to 6% in 2007 [7], Uganda remains in this category.

Reports in the scientific literature regarding the prevalence of sexual coercion vary greatly, ranging from a low ranging from a low of 5% to 20% among adolescents in northern Thailand, young people and adults in Australia; married and unmarried males and females ages 10-24 in Kenya, high school students in northeastern Nigeria, and young adults in three state capitals in Brazil [8-12]; to a high of approximately 50% in other populations, including young women in the US (taken from five samples of young people), low-income African-American women, youth and young adults in Peru, youth and adults in South Africa, adolescents in Ibadan, Nigeria, young undergraduate college students in the US, and adolescents in Jamaica [1,13-19]. Earlier studies only tended to report the prevalence among women, or else showed a considerably higher prevalence of sexual coercion among women compared with men. Other research has found similar levels for both sexes, a conclusion that has been interpreted as a function of gender equity in a multinational comparative study [20]. The great variations in the observed prevalence of sexual coercion could be due to different definitions of sexual coercion, alternative methods of data collection, varying target populations (including gender, age, and socioeconomic status) and, most importantly, true variations between different contextual settings.

In view of this, there appears to be a strong need for increasing the knowledge of sexual coercion. In many parts of the world, sexual coercion has been linked to risky sexual behavior, implying that there may be common causal pathways in the psychological reaction to serious violations of an individual's integrity: A population-based study of young people in Kenya showed that the risk of having multiple partners was doubled among those who had experienced sexual coercion [10]. A similar finding was reported in a study from Ethiopia among women between the ages of 10 and 24. Those who had experienced sexual coercion were three times more likely to have had more than one sexual partner during the year prior to the study, in comparison with other women [4]. Another study from the Caribbean reported a seven-fold increased risk of early sexual debut among high school girls who had experienced sexual coercion, compared with those who had not had such an experience [3]. Several studies have reported an association between experience of sexual coercion and higher rates of sexually transmitted diseases (STDs), implying ensuing consequences as a result of risky sexual behavior, including inconsistent condom use [9,18]. A recent Canadian study of adolescent females in difficult social circumstances showed a link between the experience of sexual coercion and a diminished ability to communicate about sexuality and contraception [5].

Estimates of the prevalence of sexual coercion point to highs in settings with an elevated prevalence of HIV/AIDS, as in South or East Africa, although this prevalence varies considerably between different settings [4,15,18,21-23]. This observation has further highlighted a possible causal link, which could provide new insights for intervention. Since the impact of sexual coercion on subsequent sexual behavior is probably modified by socio-demographic, religious, and cultural factors, it would be useful to better understand these mechanisms, in order to improve policy formulation, reliable strategies and interventions to combat HIV/AIDS. A multicountry study of women ages 12 to 19 in four sub-Saharan countries that investigated coercion on the occasion of first sexual encounter showed that 23% of those in the Ugandan sample have experienced such coercion [22]. This was corroborated by another study of adult Ugandan women from the Rakai district, where 22% of the women surveyed reported such coercion [23]. Another study of women ages 15 to 19 from the same district found an association between the experience of coercion, on the one hand, and both inconsistent condom use and an increased level of STDs, on the other [2].

According to our knowledge there have been no studies from Uganda or any other high HIV/AIDS prevalence setting investigating the subsequent impact that experiences of sexual coercion have on a variety of risky sexual behaviors. The present study seeks to fill that gap.

The chosen definition of sexual coercion is not based on any particular theory of power relations but the attempt is made to be as straightforward as possible and the definition of "being forced to do something", i.e., to participate in a sexual situation, actively or passively, is left to the respondent, believing that the understanding of this is reasonably uniform. The study also attempts to understand some of the causal mechanisms involved so that factors that might buffer the negative impact of experience of coercion can be identified.

Its principal aim, therefore, was to investigate the effect that the experience of sexual coercion had upon among a sample of university students with regard to sexual debut, number of sexual partners, and inconsistent condom use. In addition, it sought to determine whether certain individual, cultural, or social resources could protect individuals from this impact.

## Methods

### Population and setting

The study was performed at Mbarara University of Science and Technology (MUST), a public university in the city of Mbarara in southwestern Uganda. Our target population consisted of undergraduate students from the university's three faculties of medicine, science, and development studies. The sample comprised the entire undergraduate class of the university in 2005 (n = 1220 students).

The HIV prevalence in the Mbarara District in 1991 was approximately 24%, which was higher than the national prevalence of 15% in Uganda [24]. By 2001 the prevalence had declined to 10.8%. There are several possible reasons why the HIV epidemic affected southwestern Uganda more than the rest of the country [25]. Because it is a border region, sociopolitical developments have impacted the population in specific ways. During the regime of Idi Amin in the 1970s, the cross-border traffic between southwestern Uganda, Tanzania, and Rwanda increased, altering the local economy. One result was that many women resorted to prostitution as a means of earning money from the transport workers who spent the night near the border. Once the HIV epidemic had established itself, there was a sharp increase in mortality among young adults. In addition, the prevailing custom requiring widows (who may have already been HIV-positive) to marry their husband's brother or closest kinsman may have further accelerated the spread of the infection [25].

### Data collection

Data was collected by means of an 11-page self-administered questionnaire consisting of 132 questions that was distributed in lecture halls to all undergraduate students at MUST. In order to reach all students, the questionnaires were distributed over the course of two days.

Students were orally informed beforehand about the purpose of the questionnaire and were given instructions for filling it out. A consent form on the front page also contained a written explanation and justification of the project to be signed by the students as acknowledgement of being informed and agreeing to participate. Contact details for the principal investigator and a research assistant were provided in the event any questions or personal concerns arose while answering the questions. The research staff ensured that the room was silent while students were engaged in filling out the questionnaires, so that each person could work in private. The consent forms and the questionnaires (the latter containing no identifying information) were collected separately and placed in different boxes in the front of the room. A total of 980 students, representing 80% of the undergraduate students at the university, completed the questionnaires.

The questionnaire assessed lifestyle factors, such as alcohol consumption, drug use, and smoking habits; love and sex; social relations, participation, and social capital; mental health, self-rated health and social and demographic factors, such as area of origin, socioeconomic status (SES), religious affiliation, and the role of religion in one's family. The research project was approved by the Institutional Ethics Review Committee at Mbarara University of Science and Technology.

### Definition of variables

#### Background variables

*Area of origin *was dichotomized into "rural" or "urban/peri-urban or small town".

*Educational level of head of household *during childhood was dichotomized so that "did not finish primary school" and "completed primary school' were coded as "low" and any education above that was classified as "high".

*The role of religion in the family *while growing up was dichotomized so that "religion played a big role" and "religion was relatively important" coded as "major role"; and "religion was not so important" and "religion was not important at all" coded as "minor role".

*Age *was arbitrarily divided into two groups at the upper tertile: "younger" ≤ 23 years old and "older" > 23 years old. *Sex *was classified as male or female.

*Mental health*, The Hopkins Symptom Checklist (HSCL-25) was used to assess mental health. It consists of 15 items assessing symptoms of depression and 10 assessing symptoms of anxiety during the previous week [26]. In addition, we included the 10 item psychoticism sub-scale from the Symptom Checklist-90 (SCL-90) in order to assess the extent to which symptoms of this nature may have been present during the previous week [27]. Each item HSCL-25 item was rated from 1-4 to indicate a range from "not at all" to "extremely". Although the SCL-90 uses a self-reporting five-point scale, we rated its 10 items on a reduced scale of 1-4 in order to assure homogeneity with the responses for anxiety and depression. Both the HSCL-25 and the SCL-90 have been previously employed and validated in different cultural contexts in Africa [27-30]. Mean total mental health scores were calculated based on a student's total score for each of the measures, divided by the number of items the student answered. We then dichotomized the scores into "high" and "low" (indicating poor and good mental health, respectively) by calculating the median-split between the total scores for each measure.

*Trust in others *was measured on the basis of answers to four questions commonly used in epidemiological studies [31], namely, "Most people would take advantage of you if they had an opportunity", "Most people try to be fair", "You can trust most people", and "You cannot be careful enough when dealing with other people". The response alternatives were "I do not agree at all", "I do not agree", "I agree", or "I agree completely". They were accordingly assigned values from 1 to 4 (the scoring of the first and last items were reversed), yielding a maximum total score of 16. Based on the median score, the variable was dichotomized into "high trust" (above the median) or "low trust" (below the median).

*Consumed alcohol on latest occasion of sexual intercourse *was coded as "yes" or "no".

#### Experience of sexual coercion

We decided to use an instrument which has been tried in a large population study on sexual behavior, performed in Sweden in the mid-1990s [32], because it seemed feasible for respondents of all ages. The implied definition of sexual coercion was, therefore, not based on any particular theory of power relations but we sought to make it as straightforward as possible; and the definition of "being forced to do something" i.e., to participate in a sexual situation, actively or passively (as defined by the items of the instrument) is left to the respondent, believing that the understanding of this is reasonable uniform, at least at a general operational level.

Thus, experience of sexual coercion was based on a response of "yes" to any of the following questions: "You have been *forced *to show your sexual organ", "Someone has *forced *you to let them touch your sexual organ", "Someone *forced *you to let them suck or lick your sexual organ", "Someone has *forced *you to let them show you their own sexual organ", "You have been *forced *to watch someone masturbate", "You have been *forced *to masturbate someone", "You have been *forced *to take part in oral sex or to lick someone's sexual organ", "You have been *forced *to take part in sexual intercourse with the penis in the vagina, or someone has inserted an object into your vagina", or "You have been *forced *to pose for a sex photo or sex film". In the absence of any affirmative answer to the above questions, and with an affirmative answer to the question "You have not been *forced *into any of these", the individual was classified as unexposed to sexual coercion. These questions have been validated in previous studies [32].

### Dependent variables

#### Sexual behavior variables

*Previously had sex *was coded as "yes" or "no", based on the answer to the question: "Have you ever had sexual intercourse?"

*Number of lifetime sexual partners *was ascertained by the response to "How many sexual partners have you had altogether?" The variable was dichotomized so that ≥ 3 was coded as "high" and < 3 as "low".

*Condom use on latest occasion of sexual intercourse *was determined by asking "Did you use any method of avoiding sexually transmitted diseases on your latest occasion of sexual intercourse?" The response alternatives were dichotomized by classifying "yes, condom" as "consistent condom use" and "no", or "yes, other" as "inconsistent condom use".

### Statistical analysis

Sample size was given since we assessed all the students at the university, but a formal check revealed that in most analyses a 75% increase of risk could be ascertained at 80% probability. This could not exclude the risk of not being able to detect some true effects of moderate size.

The statistical analyses were done with SPSS Version 16.0. Logistic regressions were performed to calculate the crude odds ratios (OR) with 95% confidence intervals (CI) for the effect of background and consumption of alcohol on latest occasion of sexual intercourse, trust in others, scores of mental health, and experience of sexual coercion on "previously had sex", "number of lifetime sexual partners", and "condom use on latest occasion of sexual intercourse".

Multivariate logistic regression (OR with 95% CI) was used to investigate the association between sexual coercion and sexual behavior and was stepwise adjusted for age, gender, educational level of household, and role of religion and trust in others.

Effect modification between variables was calculated by means of dummy variables, as proposed by Rothman [33], according to whom a synergistic effect is present when a dependent variable has a greater impact on an outcome in the presence of a third variable (i.e., the association becomes "more than additive").

## Results

Approximately two-thirds of the sample consisted of male students, and about two-thirds were under the age of 24 (Table 1). Three-fourths of the student body came from homes headed by a person with secondary schooling or beyond. The majority of the students grew up in homes where religion played a major role. Almost one-third (31.1%) of all the students reported that they had experienced sexual coercion at some point in their lives. Fifty-nine percent had debuted sexually; 39.0% had had more than two sexual partners; and 17.3% reported inconsistent condom use. There were some gender differences, i.e., the females were somewhat younger; higher percentage of females than males grew up in a family with a higher educational level; fewer females had debuted sexually and at a higher age than male students; females also tended to have had fewer lifetime sexual partners, but had a higher rate of consistent condom use. However, regarding experience of sexual coercion, the reported prevalence was very similar between the sexes: 33.1% for females and 29.9% for males.

**Table 1:** Prevalence of socio-demographic factors, trust in others, mental health, alcohol use, experience of sexual coercion and sexual behavior factors

	All		Male		Female	
	n	%	n	%	n	%
*Sex*						
Male	633	64.6				
Female	347	35.4				
*Age*						
Younger ≤ 23	628	65.6	378	60.6	250	75.1
Older ≥ 24	329	34.4	246	39.4	83	24.9
Missing	(23)					
*Educational level of head of household*						
≤ Primary	235	25.5	414	69.0	274	84.8
> Primary school	688	74.5	186	31.0	49	15.2
Missing	(57)					
*Importance of religion*						
Major role	542	55.9	337	53.8	205	59.8
Minor role	427	44.1	289	46.2	138	40.2
Missing	(11)					
*Trust in others*						
High	544	60.2	348	59.8	196	60.9
Low	360	39.8	234	40.2	126	39.1
Missing	(76)					
*Consumed alcohol on latest occasion of sexual intercourse*^2^						
Yes	122	23.9	83	36.7	39	44.3
No	388	76.1	143	63.3	49	55.7
Missing	(22)					
*Mental health*						
High score	468	49.7	297	49.0	171	51.0
Low score	473	50.3	309	51.0	164	49.0
Missing	(39)					
*Experience of sexual coercion*						
Yes	246	31.1	153	29.9	93	33.1
No	546	68.9	358	70.1	188	66.9
Missing	(188)					
*Previously had sex*						
Yes	532	59.0	376	62.9	156	51.3
No	370	41.0	222	37.1	148	48.7
Missing	(78)					
*Age at sexual debut*						
≤ 18 = low	262	51.2	199	55.0	63	42.0
> 18 = high	250	48.8	163	45.0	87	58.0
Missing	(20)					
*Number of lifetime sexual partners*^1^						
1-2 = low	293	61.0	180	54.1	113	76.9
≥ 3 = high	187	39.0	153	45.9	34	23.1
Missing	(52)					
*Condom use at latest occasion of sexual intercourse*^1^						
Consistent	424	82.7	306	85.2	118	76.6
Inconsistent	89	17.3	53	14.8	36	23.4
Missing	(19)					

^1^Only analyzed among individuals who had had sexual intercourse

^2 ^Only analyzed among individuals who drank alcohol

Table 2 and 3 shows the pattern of impact of socio-demographic factors, social capital, alcohol consumption habits, and level of mental health on the four sexual behaviors studied. The older age group also contained a greater proportion of those who had previously had sex, which could be expected, but their age of sexual debut was higher than their younger fellow students by a statistically significant amount. There were some gender differences regarding the impact of age, however only one of those with discordant statistical significance, the older male group had a lower risk of early sexual debut.

**Table 2:** Association (Odds Ratios, 95% Confidence Intervals) between socio-demographic factors, trust in others, mental health, alcohol use, experience of sexual coercion and sexual behavior

	**Previously had sex**			**Low age of sexual debut**		
	
	**All**	**Male**	**Female**	**All**	**Male**	**Female**
	
*Sex*						
Female	1 (ref)			1 (ref)		
Male	1.6 (1.1-2.1)			1.7 (1.1-2.1)		
*Age*						
Younger	1 (ref)	1 (ref)	1 (ref)	1 (ref)	1 (ref)	1 (ref)
Older	2.1 (1.6-2.8)	1.9 (1.3-2.7)	2.2 (1.3-3.8)	0.7 (0.5-0.99)	0.6 (0.4-0.9)	0.9 (0.4-1.7)
*Educational level of head of household*						
High	1 (ref)	1 (ref)	1 (ref)	1 (ref)	1 (ref)	1 (ref)
Low	1.0 (0.8-1.4)	0.9 (0.6-1.3)	1.1 (0.6-2.2)	1.1 (0.7-1.7)	0.96 (0.6-1.5)	1.2 (0.5-3.0)
*Role of religion*						
Major role	1 (ref)	1 (ref)	1 (ref)	1 (ref)	1 (ref)	1 (ref)
Minor role	1.2 (0.96-1.6)	1.4 (0.98-1.9)	1.0 (0.6-1.5)	1.7 (1.2-2.4)	1.7 (1.1-2.5)	1.6 (0.8-3.1)
*Trust in others*						
High	1 (ref)	1 (ref)	1 (ref)	1 (ref)	1 (ref)	1 (ref)
Low	1.0 (0.7-1.3)	1.3 (0.9-1.9)	0.6 (0.3-0.9)	1.4 (0.99-2.1)	1.4 (0.9-2.1)	1.4 (0.7-2.9)
*Mental health*						
Low score	1 (ref)	1 (ref)	1 (ref)	1 (ref)	1 (ref)	1 (ref)
High score	1.4 (1.1-1.9)	1.5 (1.04-2.1)	1.4 (0.9-2.2)	1.5 (1.06-2.1)	1.6 (1.1-2.5)	1.3 (0.7-2.5)
*Consumed alcohol on latest occasion of sexual intercourse*^1^						
No				1 (ref)	1 (ref)	1 (ref)
Yes				2.0 (1.3-3.2)	3.2 (1.7-6.2)	1.8 (0.7-4.8)
*Experience of* *sexual coercion*						
No	1 (ref)	1 (ref)	1 (ref)	1 (ref)	1 (ref)	1 (ref)
Yes	1.5 (1.1-2.1)	1.5 (0.98-2.2)	1.7 (1.02-3.0)	2.3 (1.6-3.5)	2.2 (1.3-3.5)	3.3 (1.6-6.9)

^1 ^only analyzed among those who drank alcohol

**Table 3:** Association (Odds Ratios, 95% Confidence Intervals) between socio-demographic factors, trust in others, mental health, alcohol use, experience of sexual coercion and sexual behavior

	**High number of lifetime sexual partners**			**Inconsistent condom use**		
	
	**All**	**Male**	**Female**	**All**	**Male**	**Female**
	
*Sex*						
Female	1 (ref)			1 (ref)		
Male	2.8 (1.8-4.3)			0.6 (0.4-0.9)		
*Age*						
Younger	1 (ref)	1 (ref)	1 (ref)	1 (ref)	1 (ref)	1 (ref)
Older	1.3 (0.9-1.9)	1.1 (0.7-1.7)	1.8 (0.8-4.1)	1.3 (0.8-2.1)	1.2 (0.6-2.1)	2.2 (0.99-4.7)
*Educational level of head of household*						
High	1 (ref)	1 (ref)	1 (ref)	1 (ref)	1 (ref)	1 (ref)
Low	1.4 (0.9-2.1)	1.2 (0.8-2.0)	1.0 (0.3-2.9)	1.0 (0.6-1.7)	1.2 (0.7-2.3)	0.8 (0.2-2.4)
*Role of religion*						
Major role	1 (ref)	1 (ref)	1 (ref)	1 (ref)	1 (ref)	1 (ref)
Minor role	1.6 (1.1-2.3)	1.3 (0.9-2.0)	2.2 (1.01-4.8)	1.1 (0.7-1.7)	1.0 (0.6-1.8)	1.4 (0.7-3.1)
*Trust in others*						
High	1 (ref)	1 (ref)	1 (ref)	1 (ref)	1 (ref)	1 (ref)
Low	1.1 (0.7-1.6)	1.1 (0.7-1.8)	0.8 (0.3-1.8)	1.7 (1.03-2.7)	1.6 (0.9-3.0)	2.0 (0.9-4.5)
*Mental health*						
Low score	1 (ref)	1 (ref)	1 (ref)	1 (ref)	1 (ref)	1 (ref)
High score	1.2 (0.7-1.6)	1.3 (0.8-2.0)	1.1 (0.5-2.4)	1.2 (0.7-1.9)	1.1 (0.6-2.0)	1.3 (0.6-2.7)
*Consumed alcohol on latest occasion of sexual intercourse*^1^						
No	1 (ref)	1 (ref)	1 (ref)	1 (ref)	1 (ref)	1 (ref)
Yes	3.8 (2.3-6.2)	7.2 (3.5-15.0)	2.4 (0.8-7.0)	0.9 (0.5-1.6)	0.9 (0.4-2.1)	1.3 (0.5-3.9)
*Experience of* *sexual coercion*						
No	1 (ref)	1 (ref)	1 (ref)	1 (ref)	1 (ref)	1 (ref)
Yes	1.6 (1.1-2.5)	1.9 (1.2-3.1)	1.6 (0.7-3.7)	1.1 (0.6-1.8)	1.3 (0.7-2.5)	0.7 (0.3-1.7)

^1 ^only analyzed among those who drank alcohol

The educational level of the head of the household where a student grew up did not show any strong association with any of the outcomes studied. However, students from those households where religion played a minor role had a greater likelihood of having debuted sexually at a younger age and of having had more sexual partners than those from households in which religion played a major role. Trust in others, a dimension of social capital, was associated with condom use, so that students with low trust in others reported inconsistent condom use to a greater extent than those with high trust in others. Some gender differences were observed; religion playing a minor role was more clearly associated with a higher number of lifetime sexual partners, and low trust in others with a lower risk of sexual debut in females, as compared to males.

Elevated total mental health scores (poor mental health) was associated with a greater likelihood of having debuted sexually, and having done so at an earlier age than those with a low mental health score, but there were no clear associations between this variable and reporting higher number of sexual partners or inconsistent condom use. Having consumed alcohol on latest occasion of sexual intercourse was associated with a doubled risk of having had an early sexual debut (OR 2.0, 95% CI: 1.3-3.2) and an almost quadrupled risk of having had many sexual partners (OR 3.8, 95% CI: 2.3-6.2). However, the risk for inconsistent condom use was not higher in this group. Gender differences were noted for some of these associations: a high score of total mental health factors and consumption of alcohol during latest sexual intercourse were associated with higher risk of low age at sexual debut among males, but not in females. Alcohol consumption was also associated with higher risk for having higher number of sexual partners among males, compared with females.

Among those who had experienced sexual coercion, a 50% higher likelihood of having debuted sexually was noted (OR 1.5, 95% CI: 1.1-2.1), along with a more than doubled risk of having done so at an early age (OR 2.3, 95% CI: 1.6-3.5). Moreover, individuals who had experienced sexual coercion also reported a higher number of sexual partners (OR 1.6, 95% CI: 1.1-2.5). However, their experience seemed unrelated to inconsistent condom use (OR 1.1, 95% CI: 0.6-1.8). The experience of sexual coercion seemed to have a stronger association with a high number of sexual partners among males than females.

Table 4 shows the result of the multivariable logistic regression analyses performed to account for possible confounding regarding the association between the main exposure in this study, experience of sexual coercion, and the four sexual behaviors. The same potential confounders were used for all four outcomes studied (sexual behaviors). They were introduced in the same order in three groups in order to facilitate the comparison of the impact of the different factors.

**Table 4:** Association (Odds Ratios, 95% Confidence Intervals) between experienced sexual coercion and sexual behavior in a sample of Ugandan university students.

**Sexual behavior**	**Model 1**			**Model 2**		
	**(adjusted for age and educational level of head of household)**			**(adjusted for age, educational level of head of household, role of religion and trust in others)**		
	**All**	**Male**	**Female**	**All**	**Male**	**Female**
	
*Previously had sex*						
Experience of sexual coercion	1.7 (1.2-2.4)	1.7 (1.1-2.6)	1.6 (0.9-3.0)	1.6 (1.1-2.3)	1.6 (1.004-2.5)	1.7 (0.9-3.0)
Minor role of religion				1.3 (0.9-1.8)	1.4 (0.9-2.1)	1.1 (0.6-2.0)
Low trust in others				0.9 (0.6-1.2)	1.1 (0.6-2.0)	0.5 (0.3-0.9)
						
*Low age of sexual debut*						
Experience of sexual coercion	2.5 (1.6-3.9)	2.4 (1.4-4.0)	3.2 (1.4-7.1)	2.4 (1.5-3.7)	2.3 (1.3-3.9)	3.0 (1.3-6.9)
Minor role of religion				1.4 (0.9-2.2)	1.4 (0.8-2.2)	1.7 (0.7-3.8)
Low trust in others				1.4 (0.9-2.1)	1.1 (0.7-1.9)	2.2 (0.9-5.2)
						
*High number of lifetime sexual partners*						
Experience of sexual coercion	1.9 (1.2-3.0)	2.2 (1.3-3.7)	1.3 (0.5-3.6)	1.9 (1.2-3.0)	2.1 (1.2-3.7)	1.4 (0.5-4.0)
Minor role of religion				1.7 (1.1-2.7)	1.4 (0.8-2.4)	3.5 (1.2-10.0)
Low trust in others				1.0 (0.6-1.6)	1.01 (0.6-1.7)	0.9 (0.3-2.5)
						
*Inconsistent condom use*						
Experience of sexual coercion	1.0 (0.6-1.7)	1.3 (0.6-2.5)	0.7 (0.3-1.7)	0.9 (0.5-1.5)	1.2 (0.6-2.5)	0.6 (0.2-1.5)
Minor role of religion				0.9 (0.5-1.6)	0.7 (0.4-1.4)	1.3 (0.5-3.2)
Low trust in others				2.0 (1.1-3.4)	1.3 (0.7-2.7)	3.6 (1.4-9.3)

Results of multivariate logistic regression analyses.

The association between experience of sexual coercion and previously had sex (OR 1.6, 95% CI: 1.1-2.3) and low age of sexual debut (OR 2.4, 95% CI: 1.5-3.7) persisted after adjusting for the potential confounders (age, educational level of head of household, role of religion, and trust in others). Gender differences were marginal.

The association between experience of sexual coercion and having many sexual partners remained statistically significant when the three socio-demographic variables were introduced into the model (OR 1.9, 95% CI: 1.2-3.0). Moreover, when religion and trust in others were introduced, the significant association persisted (OR 1.9, 95% CI: 1.2-3.0). However, when mental health score and alcohol consumption pattern were introduced (data not shown), the association was weakened with 30%, which in this case most likely represents a considerable risk of over-adjustment. There was a notable difference between males and females in that the experience of sexual coercion was strongly associated with a high number of sexual partners among females, as opposed to males.

In Table 5, the effect modification by gender regarding the impact of experience of sexual coercion on all the studied sexual behaviors was investigated in a more formal way using the "more than additivity" criterion. The effect of experience of sexual coercion was not modified by gender for the sexual behaviors with the exception that gender seemed to modify the effect of experience of sexual coercion on consistent condom use, so that women with this experience tended to have a lower risk for unprotected sex, while the pattern was in the opposite direction for men.

**Table 5:** Analysis of effect modification between gender and experienced sexual coercion regarding sexual behavior, presented as adjusted OR with 95% CI

	**Previously had sex**	
	
	All	
	n (%)	Odds Ratios (CI)
Male/no coercion	358 (45.2)	1 (ref)
Male/coercion	153 (19.3)	1.5 (0.98-2.2)
Female/no coercion	188 (23.7)	0.6 (0.4-0.8)
Female/coercion	93 (11.7)	1.0 (0.6-1.6)
(Missing)	(188)	
Total	980	
	**Low age of sexual debut**	
	
	All	
	n (%)	Odds Ratios (CI)
Male/no coercion	210 (47.8)	1 (ref)
Male/coercion	100 (22.8)	2.2 (1.3-3.5)
Female/no coercion	79 (18.0)	0.5 (0.3-0.8)
Female/coercion	50 (11.4)	1.5 (0.8-2.8)
(Missing)	(93)	
Total	532	
	**High numbers of lifetime sexual partners**	
	
	All	
	n (%)	Odds Ratios (CI)
Male/no coercion	190 (46.2)	1 (ref)
Male/coercion	94 (22.9)	1.9 (1.2-3.1)
Female/no coercion	77 (18.7)	0.3 (0.2-0.6)
Female/coercion	50 (12.2)	0.5 (0.3-1.03)
(Missing)	(121)	
Total	532	
	**Inconsistent condom use**	
	
	All	
	n (%)	Odds Ratios (CI)
Male/no coercion	205 (46.9)	1 (ref)
Male/coercion	101 (23.1)	1.3 (0.7-2.5)
Female/no coercion	79 (18.1)	2.3 (1.2-4.3)
Female/coercion	52 (11.9)	1.7 (0.8-3.7)
(Missing)	(95)	
Total	532	

In Table 6 we chose to present separately for each gender the results of analysis of interaction between experience of sexual coercion and three selected exposure variables (mental health score, role of religion, and trust in others) regarding the risk of having had many sexual partners. The mental health score showed a clear tendency of modifying the effect of experience of sexual coercion, so that sexual coercion was only associated with a higher number of sexual partners when the mental health score was high (poor mental health). This pattern was strongest among male students.

**Table 6:** Analysis of effect modification between mental health, role of religion and trust in others on the one hand and sexual coercion on the other, regarding sexual behavior presented as adjusted OR with 95% CI

**High numbers of sexual partners**	**Female**		**Male**	
	**n (%)**	**Odds Ratios (95% CI)**^**1**^	**n (%)**	**Odds Ratios** **(95% CI)**^**1**^
	
*Mental health score/Experience of sexual coercion*				
Low mental health score/No coercion	46 (37.7)	1 (ref)	101 (36.9)	1 (ref)
Low mental health score/Coercion	14 (11.5)	0.7 (0.1-3.9)	27 (9.9)	1.1 (0.4-2.5)
High mental health score/No coercion	29 (23.8)	0.8 (0.2-2.8)	81 (29.6)	1.1 (0.6-1.9)
High mental health score/Coercion	33 (27.0)	1.7 (0.9-5.9)	65 (23.7)	2.6 (1.4-5.0)
(Missing)	(34)		(102)	
Total	156		376	
				
*Role of religion/Experience of sexual coercion*				
Major role of religion/No coercion	47 (37.9)	1 (ref)	102 (36.6)	1 (ref)
Major role of religion/Coercion	29 (23.4)	1.1 (0.3-4.2)	46 (16.5)	1.4 (0.7-2.8)
Minor role of religion/No coercion	30 (24.2)	2.1 (0.6-7.2)	84 (30.1)	1.1 (0.6-2.0)
Minor role of religion/Coercion	18 (14.5)	4.8 (1.3-17.7)	47 (16.8)	2.9 (1.4-6.0)
(Missing)	(32)		(97)	
Total	156		376	
				
*Trust in others/Experience of sexual coercion*				
High trust in others/No coercion	53 (45.7)	1 (ref)	112 (43.8)	1 (ref)
High trust in others/Coercion	29 (25.0)	0.7 (0.2-2.5)	41 (16.0)	2.0 (0.96-4.1)
Low trust in others/No coercion	17 (14.7)	0.4 (0.1-2.0)	55 (21.5)	0.9 (0.5-1.7)
Low trust in others/Coercion	17 (14.7)	1.8 (0.5-6.4)	48 (18.8)	2.4 (1.2-4.7)
(Missing)	(40)		(120)	
Total	156		376	

^1 ^Adjusted for age

Role of religion also seemed to modify the effect of experience of sexual coercion, so that it was only associated with a higher number of sexual partners among those who had grown up in families where religion did not play a major role. This pattern was similar among males and females.

Trust in others tended to modify the effect of experience of sexual coercion in females, so that an increased risk for many sexual partners was only observed among those with low trust in others. Such a pattern was not statistically significant among male students.

## Discussion

The results of our study showed that the experience of sexual coercion was associated with riskier sexual behavior among students of both sexes in our sample. The association with early sexual debut seemed particularly robust, and remained statistically significant, even when controlled for potential socio-demographic confounders. There was also some indication from our analyses that alcohol consumption habits and mental health were mediating variables between having experienced sexual coercion and early sexual debut as well as having many sexual partners. However, there was no obvious support in our results for an association between exposure to sexual coercion and inconsistent condom use. These results showed similar patterns for men and women, with a few exceptions.

We also found some evidence that factors like mental health status, religious commitment, and social capital modified the effect of exposure to sexual coercion and the likelihood of having had many sexual partners. It appeared that having a good mental health status, coming from a family where religion played a major role, or having high trust in others seemed to protect the individuals who had been exposed to sexual coercion from the risk of having many sexual partners, in comparison to others who lacked the characteristics cited.

In our study, the prevalence of sexual coercion appeared to be higher than reported in previous studies in Uganda [34]. However, it is difficult to make direct comparisons because of the different assessment methods, target groups, and the time periods involved. The instrument we used was developed and validated in a large national survey of sexuality in Sweden, and is considered effective in assessing coercion events of significance for those who report exposure of all ages [32]. It also provided some kind of reference although performed in a different setting. In fact, the contrasts per se may be valuable for generating hypotheses. Admittedly, the perception of "being forced to do something" is likely to be deeply rooted in the local cultural context, but we choose to acknowledge this in the interpretation of the findings, rather than develop a context specific instrument that might miss the opportunity to make comparisons between different settings, which is one of the main lines of current research in the area, i.e., comparative studies of sexual violence between countries or settings using a standardized instrument.

According to our findings, 33.1% of the females and 29.9% of the males who participated in our study experienced significant events of sexual coercion. To our knowledge, this study represents the first time that the prevalence of sexual coercion has been assessed in a group of young males in Uganda. It has been claimed that such prevalence is considerably lower among males than females in societies with substantial gender inequity [20]. Such a hypothesis is not supported by our data for Uganda, a country where gender equity is conspicuously absent. This would appear to indicate that different causal mechanisms are operative than the ones suggested in the earlier study. However, our data does not support any further speculation concerning this issue.

It has also been suggested that the impact of experience of sexual coercion differs from males to females [14]. However, this hypothesis also finds little support in our analyses. Also, we could corroborate previous findings that the experience of sexual coercion is associated with two risky sexual behaviors: early sexual debut and having many sexual partners [1-5]. Reports in the studies just cited of an association with inconsistent condom use could not be confirmed. However, the results from this study cannot claim to represent the entire group of students in Uganda and might differ from other universities situated in different cultural and geographical areas.

Some authors have claimed that the Population Attributable Risk (PAR) for experience of sexual coercion regarding certain types sexual behavior is on the order of 25% [35]. The implication is that if sexual coercion could be abolished altogether, those sexual behaviors would decrease by 25% in the target population. Using the estimated prevalence of experience of sexual coercion found in our study and the estimated increased risky sexual behavior due to this factor, we arrive at estimates of the PAR regarding experience of sexual coercion in our target population on the order of 20% to 25% for early sexual debut and having many sexual partners. In our view, this demonstrates a considerable prevention potential regarding STDs and HIV/AIDS in this setting, provided that effective means of intervention may be introduced.

In this perspective, we feel that our findings regarding those factors that could buffer the impact of sexual coercion on risky sexual behavior (particularly having many sexual partners) might be of considerable interest. Our analyses suggest that such protective factors exist on three levels: the individual (good mental health status), the immediate social environment (social capital in terms of high trust in others), and the socio-cultural (high religious commitment in the family of origin). Thus, future policy formulations and implementation strategies may well benefit from these findings.

## Study limitations

This study's cross-sectional design does not allow one to judge the direction of causation between the main exposure, experience of sexual coercion, and sexual behaviors. Sexual coercion could be of two principal types: coercion directed toward a young person by an older individual or an adult (i.e., before the victim becomes a university student), or coercion directed towards a university student by a sexual or dating partner who is a fellow student or another adult (sometimes considerably older, as in cases of trans-generational sex). Since we lacked information regarding the type of coercion the students in our sample experienced, this circumstance made determining the causal direction problematical. An additional difficulty is presented by the possibility that the outcomes "sexual debut" or "early sexual debut" resulting from sexual coercion may have happened before entry into the university.

Selection bias may be another issue of concern. Although the general response rate was quite high (at least compared with other voluntary surveys using questionnaires, targeting healthy and relatively unselected populations) at 80%, of the full target group, non-response regarding the questions assessing experience of sexual coercion was at the level of an additional 20%. One might assume that the proportion of those exposed to sexual coercion was higher among non-responders than among responders, since this could be considered a stigmatizing experience that some individuals would be reluctant to disclose. As socially undesirable behaviors are generally assumed to be underreported in surveys as well, it is not unlikely that "exposed cases" (i.e., individuals exposed to sexual coercion and subsequently engaging in sexually risky behaviors) are overrepresented among the non-responders. If this is the case, the associations between those phenomena are underestimated in our study.

Dependent misclassification could also be an issue in cases where individuals who experienced sexual coercion are more prone to report risky sexual behavior than those without such experience, or vice-versa. However, we cannot think of any compelling arguments that would support this assumption.

The possible confounding effect of a number of socio-demographic variables and other known determinants of risky sexual behavior was controlled for in the multivariate analysis. The inclusion of a high number of variables in logistic regression models could result in "over-controlling", since some of the variables do not completely fulfill the criteria of not being in the same causal chain as the main exposure and the outcomes. This would lead to an underestimation of the associations. Conversely, failure to control for important variables not assessed in this study might lead to a corresponding overestimation--a scenario that, by definition, is not amenable.

Since we chose to target all the students at the university where our study was performed, the sample size was given. We calculated that differences in effect by exposure versus non-exposure were on the order of 50% to 75%, and could be demonstrated by a probability of at least 80% regarding the main analyses (the probability was lower for the analyses concerning effect modification). This would admit the possibility of "type two" errors, i.e., accepting the null hypothesis where it should have been rejected.

## Conclusion

This study demonstrated a clear association between the experience of sexual coercion and the subsequent practice of risky sexual behavior among a sample of Ugandan university students. Since almost one-third of all students who responded to our questionnaire reported such experiences, addressing this situation would appear to be of considerable impact in preventing STDs (particularly HIV/AIDS) among young people in similar settings. These findings have implications for policy formulation and the question of children's rights, as well as for university policies on student conduct. Moreover, they would also affect the provision of services for sexual and reproductive health and rights (SRHR) among young individuals.

## Competing interests

The authors declare that they have no competing interests.

## Authors' contributions

AA and POO designed the study and performed the analysis of data. AA collected the data and drafted the manuscript. KOP participated in the analysis of data. All authors read and approved the final manuscript.

## Pre-publication history

The pre-publication history for this paper can be accessed here:

http://www.biomedcentral.com/1471-2458/11/527/prepub
